# The activation of microRNA-520h–associated TGF-β1/c-Myb/Smad7 axis promotes epithelial ovarian cancer progression

**DOI:** 10.1038/s41419-018-0946-6

**Published:** 2018-08-29

**Authors:** Jing Zhang, Wenxue Liu, Fangqian Shen, Xiaoling Ma, Xiaorui Liu, Fuju Tian, Weihong Zeng, Xiaowei Xi, Yi Lin

**Affiliations:** 1 International Peace Maternity & Child Health Hospital, School of Medicine, Shanghai Jiao Tong University, 910 Hengshan Road, Shanghai, 200030 China; 2 Department of Obstetrics and Gynecology, Shanghai General Hospital, School of Medicine, Shanghai Jiao Tong University, 85 Wujin Road, Shanghai, 200080 China

## Abstract

Among the gynaecological cancers, epithelial ovarian cancer (EOC) has the highest lethality because of the high incidence of tumour progression and metastasis. Exploration of the detailed mechanisms underlying EOC metastasis and the identification of crucial targets is important to better estimate the prognosis and improve the treatment of this disease. The present study aimed to identify the role of miR-520h in the prognosis of patients with EOC, and the mechanisms of its involvement in EOC progression. We showed that miR-520h was upregulated in 116 patients with EOC, especially in those with advanced-stage disease, and high miR-520h expression predicted poor outcome. Furthermore, ectopic expression of miR-520h enhanced EOC cell proliferation, migration and invasion, and induced epithelial–mesenchymal transition in vitro and in vivo. miR-520h promoted EOC progression by downregulating Smad7, and subsequently activating the TGF-β signalling pathway. Most importantly, TGF-β1 stimulation increased miR-520h expression in EOC cells by upregulating its transcription factor c-Myb. In conclusion, we described the role of the TGF-β1/c-Myb/miR-520h/Smad7 axis in EOC metastasis, and highlighted the possible use of miR-520h as a prognostic marker for EOC.

## Introduction

Ovarian cancer is the leading cause of cancer-related death among the gynaecological cancers^1,2^. Epithelial ovarian cancer (EOC), the most common pathological type, accounts for ~90% of all ovarian cancer^3^. Although surgical techniques have improved and new targeted drugs are being used clinically, patients with advanced EOC only have 45% expected 5-year overall survival (OS), mainly because of frequent tumour metastasis^4–6^. EOC metastasis is complex, involving oncogene activation and tumour suppressor inactivation^7–9^. To date, the mechanisms underlying these processes remain unclear.

MicroRNAs (miRNAs) are non-coding 20–24 nucleotide long RNAs that can suppress gene expression by binding to the 3′ untranslated region (3′ UTR) of their target mRNAs and can be detected in the blood^10,11^. Accumulating evidence reveals that miRNAs play a vital role in tumour biology, including tumour proliferation and metastasis^12–15^. Additionally, aberrant miRNA expression might be involved in EOC progression and metastasis^16–18^. The miR-520 family contains several members that have been reported in human cancers. For example, miR-520d-5p enhances gastric cancer cell proliferation and survival^19^, and miR-520h is crucial for death-associated protein kinase 2 (DAPK2) regulation and breast cancer progression^20^. In addition, miR-520h could facilitate lung cancer progression^21^. Moreover, miR-520h/PP2A/NF-κB signalling could mediate metastasis in cervical cancer^22^. These evidences reveal that miR-520h plays significant roles in the progression of human cancers. However, there has been no report about miR-520h in EOC. Previously, we revealed that miR-520g could promote EOC progression and chemoresistance by downregulating DAPK2 expression^23^. Despite both miR-520g and miR-520h downregulating DAPK2 expression, and promoting cancer progression, it is unknown whether miR-520h contributes to EOC progression.

The transforming growth factor β (TGF-β) signalling pathway is a classic pathway whose over-activation contributes to tumour progression^24–26^. TGF-β signalling can induce epithelial–mesenchymal transition (EMT), which is common in tumour metastasis, and is characterised by downregulation of epithelial markers and upregulation of mesenchymal markers^27–29^. Increasing studies have suggested that miRNAs regulate the capability of TGF-β to induce EMT. miR-181a can activate TGF-β/Smad signalling and induce EMT, playing a critical role in the progression of advanced ovarian cancer^30^. miR-4775 promotes colorectal cancer invasion and metastasis by Smad7/TGF-β-mediated EMT^31^. Bioinformatics analyses showed that miR-520h is also a potential regulator of TGF-β/Smad signalling. Therefore, we decided to investigate whether miR-520h induces EOC progression via TGF-β/Smad signalling.

Here we found that miR-520h is overexpressed in EOC tissues and high miR-520h expression predicts poor prognosis in human EOC, especially in patients with disease progression. We also showed that miR-520h promotes EOC cell proliferation, migration, and invasion, and induces EMT in vitro and in vivo. Moreover, miR-520h promotes EOC progression by activating TGF-β1/Smad7 signalling. Smad7 overexpression attenuates the cancer-promoting effect of miR-520h. Importantly, in EOC, TGF-β1 increases miR-520h expression by upregulating its upstream transcription factor (TF) c-Myb. Our findings indicated that miR-520h is a novel regulator of the TGF-β1/Smad7 pathway and is a potential prognostic marker in EOC.

## Results

### miR-520h is upregulated in EOC tissues and high miR-520h expression predicts poor survival

**Fig. 1:**
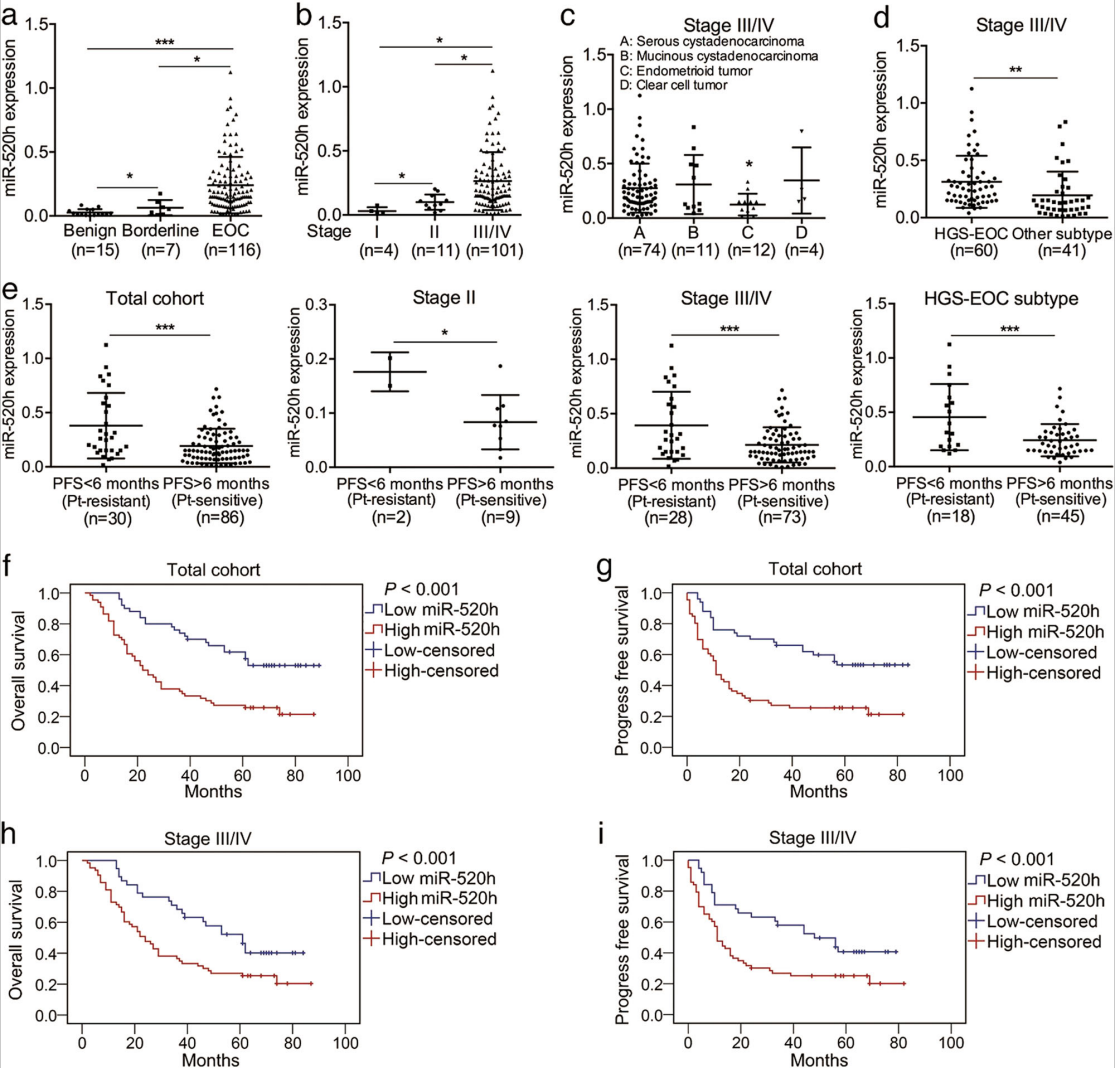
Upregulation of miR-520h promotes EOC progress and predicts poor survival. **a** Relative miR-520h levels in benign ovarian tumour, borderline ovarian tumour, and EOC tissues. **b** Relative miR-520h expression in stage I, II, and III/IV EOC tissues. **c** miR-520h levels in different pathological types of 101 cases of stage III/IV EOC. **d** Relative miR-520h levels in stage III/IV EOC tissues with high-grade serous (HGS) EOC vs. the other subtypes. **e** Relative miR-520h expression in EOC tissues of the total cohort, the stage II subgroup, the stage III/IV subgroup, and the HGS subtype with PFS < 6 months vs. PFS > 6 months. **f, g** OS (**f**) and PFS (**g**) in 116 cases of EOC analysed using Kaplan–Meier analysis with log-rank testing according to miR-520h levels. **h**, **i** Kaplan–Meier curves of OS (**h**) and PFS (**i**) in 101 cases of stage III/IV EOC based on miR-520h expression. Pt indicates platinum. *P *<* 0.05; **P *<* 0.01; ***P *<* 0.001

To explore the clinical role of miR-520h, we first evaluated miR-520h expression in human tissues. We analysed 15 benign ovarian tumours, seven borderline ovarian tumours, and 116 EOC samples, and found that miR-520h levels gradually increased from the benign to the EOC tissues (Fig. 1a). Among the 116 cases of EOC, 4 were stage I, 11 were stage II, and 101 were stage III–IV, according to the FIGO (International Federation of Gynecology and Obstetrics) staging (Supplementary Table S1). miR-520h levels in EOC gradually increased from stage I to stage III/IV (Fig. 1b). Most stage III/IV cases showed high miR-520h expression. However, a few cases (17/101) in the stage III–IV group had very low miR-520h levels. Thus, we analysed miR-520h expression among four pathological subtypes and found that the endometrioid tumour subtype had the lowest miR-520h level in both the stage III/IV subgroup and the total cohort (Fig. 1c and Supplementary Figure S1a). In clinical, high-grade-serous (HGS) EOC accounts for the majority deaths from ovarian cancer and has a high metastatic tendency^32^. We further separated HGS-EOC cases from the total cohort and compared miR-520h levels between the HGS and other subtypes. Interestingly, miR-520h expression of the HGS-EOC subtype was substantially higher than that of the other subtypes in both stage III/IV subgroup and the total cohort (Fig. 1d and Supplementary Figure S2b). Furthermore, tumours from patients who had disease progression within 6 months (progression free survival (PFS) < 6 months; clinically described as platinum resistant) showed higher expression of miR-520h compared with that in patients with PFS > 6 months (platinum sensitive) in all groups (total cohort, stage II, stage III/IV, and HGS-EOC) (Fig. 1e). The positive correlation between miR-520h expression and PFS < 6 months (platinum resistant) was further verified using Spearman’s rank analysis (*P* *<* 0.05; Supplementary Table S2–S5). Correlation analysis further indicated that high miR-520h expression in EOC tissues was positively associated with aggressive clinical features (Supplementary Table S6). Consequently, these findings suggest high level of miR-520h might be associated with EOC progression and metastasis.

Subsequent Kaplan–Meier curves showed that patients with high miR-520h levels had poorer OS and PFS compared to those with low miR-520h levels (Fig. 1f, g). To confirm the prognostic role of miR-520h, we separated stage III/IV cases from the cohort and repeated the Kaplan–Meier analysis. High miR-520h was also positively associated with the short OS and PFS of patients with stage III/IV EOC (Fig. 1h, i). Collectively, these data showed that high miR-520h expression promotes EOC progression and indicates poor prognosis.

### miR-520h promotes EOC cell proliferation and invasion, and induces EMT in vitro

**Fig. 2:**
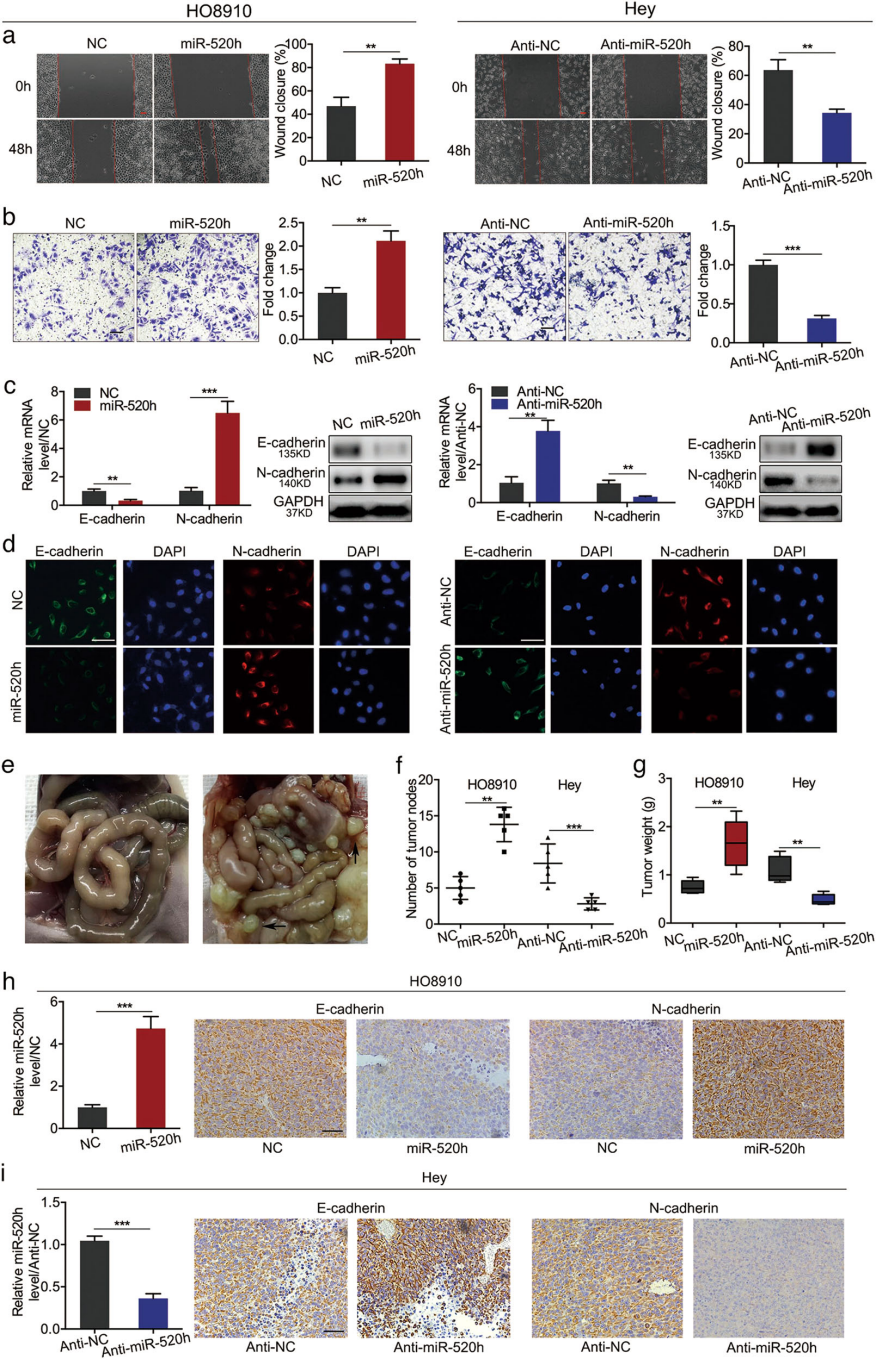
miR-520h promotes the migration and invasion of EOC cells in vitro, and tumour dissemination in vivo. **a**, **b** Effect of the overexpression or knockdown of miR-520h on wound healing (**a**) and cell invasion (**b**); magnification ×100; scale bars = 100 μm. **c** qPCR and western blotting showing changes in E-cadherin and N-cadherin levels after upregulation (in HO8910 cells) or downregulation (in Hey cells) of miR-520h. **d** Immunofluorescence detection of E-cadherin and N-cadherin after upregulation (in HO8910 cells) or downregulation (in Hey cells) of miR-520h (magnification, ×200; scale bars = 50 μm). miR-520h–overexpressing HO8910 cells, miR-520h–silenced Hey cells, and their negative control cells were injected intraperitoneally (i.p.) into 6 to 8 week-old female nude mice. The mice were killed after 3 weeks and the intraperitoneal nodules were counted. **e** Representative images of the normal control (left) and peritoneal tumour dissemination (right) in nude mice. Black arrows indicate two of the peritoneal tumour nodes. **f** Number of peritoneal tumour nodes counted using a dissecting microscope (*n* *=* 5). **g** Total weights of peritoneal tumour nodes per nude mouse (*n* *=* 5). **h**, **i** miR-520h levels and representative images of E-cadherin and N-cadherin IHC staining of peritoneal tumours derived from miR-520h–overexpressing HO8910 cells, miR-520h–silenced Hey cells, and their negative controls (magnification, ×200; scale bars = 50 μm). *P *<* 0.05; **P *<* 0.01; ***P *<* 0.001. NC negative control

To investigate the biological function of miR-520h in EOC, we measured miR-520h expression in six EOC cell lines, one benign ovarian tumour cell line, and one normal ovarian epithelial cell line. Notably, miR-520h levels were much higher in EOC cells than in the benign and normal cells (Supplementary Figure S2a). Among the EOC cell lines, we selected HO8910 (lowest miR-520h expression) and Hey (highest miR-520h expression) cells for further studies. miR-520h mimics were used to overexpress miR-520h in HO8910 cells; anti-miR-520h oligonucleotides were used to knockdown miR-520h in Hey cells (Supplementary Figure S2b and S2c). Cell Counting Kit-8 (CCK-8) assays showed that ectopic miR-520h expression significantly enhanced HO8910 cell proliferation. Furthermore, levels of the proliferation-related markers cyclin dependent kinase 6 (CDK6) and Cyclin D1 were markedly upregulated following miR-520h overexpression (Supplementary Figure S3a). By contrast, miR-520h knockdown in Hey cells had the opposite effect, as shown by CCK-8 and western blotting assays (Supplementary Figure S3b). Additionally, wound healing assays showed that miR-520h overexpression accelerated wound closure, while miR-520h knockdown delayed it (Fig. 2a). Transwell matrigel assays showed that miR-520h upregulation increased cell invasion, while miR-520h downregulation reduced it (Fig. 2b). These results indicated that miR-520h promotes EOC cell proliferation and enhances EOC metastasis in vitro. EMT plays a significant role in EOC metastasis, accompanied by loss of the cells’ epithelial phenotype, gain of a mesenchymal phenotype, and changes in EMT marker expression^33–35^. Therefore, we investigated whether miR-520h induced EMT in EOC cells in vitro. Ectopic miR-520h expression repressed the mRNA and protein expression of E-cadherin while enhancing that of N-cadherin in HO8910 cells (Fig. 2c, d). miR-520h knockdown produced the opposite results in Hey cells (Fig. 2c, d). These results indicated that miR-520h induces EMT and acts as an oncomiR in EOC cells in vitro.

### miR-520h enhances EOC cell growth and dissemination, and induces EMT in vivo

Next, we performed subcutaneous tumour xenograft assays in nude mice by injecting cells overexpressing miR-520h or knockdown miR-520h, and measuring the weight and volume of the tumours generated. Compared with the negative control, miR-520h-overexpressing or silenced cells generated larger or smaller xenografts, respectively (Supplementary Figure S3c and S3d). Subsequent quantitative real-time polymerase chain reaction (qPCR) assays of tumour xenografts verified the higher and lower expression of miR-520h in the tumours generated by injecting miR-520h-overexpressing or silenced cells, respectively, compared with that in the negative controls (Supplementary Figure S3e and S3f). Additionally, immunohistochemical (IHC) staining showed that the miR-520h-overexpression-generated larger tumours had stronger Ki-67 staining, and the opposite results were obtained for the miR520-silencing-generated smaller tumours (Supplementary Figure S3e and S3f). These results suggested that miR-520h promotes EOC cell proliferation and tumour formation in vivo.

To evaluate the miR-520h’s contribution to tumour dissemination in vivo, we injected miR-520h-overexpressing HO8910 cells, miR-520h-silenced Hey cells, or their negative controls intraperitoneally (i.p.) into nude mice. After 3 weeks, we killed the mice and counted the number of tumour nodes in the peritoneal cavity (Fig. 2e). The miR-520h-overexpressing group had more and heavier disseminated nodes per mouse, while the miR-520h-silenced group had smaller and lighter metastatic nodes, both compared with those in their negative controls (Fig. 2f, g). These data indicated that miR-520h promotes EOC cell metastasis in vivo. Consistent with the in vitro assays described above, miR-520h-overexpressing metastatic nodes had weaker E-cadherin and stronger N-cadherin staining (Fig. 2h); the opposite results were obtained for the miR-520h-silenced group (Fig. 2i). These findings further suggested that miR-520h enhances EOC cell growth and dissemination, and induces EMT in vivo.

### miR-520h downregulates Smad7 in vitro and in vivo

**Fig. 3:**
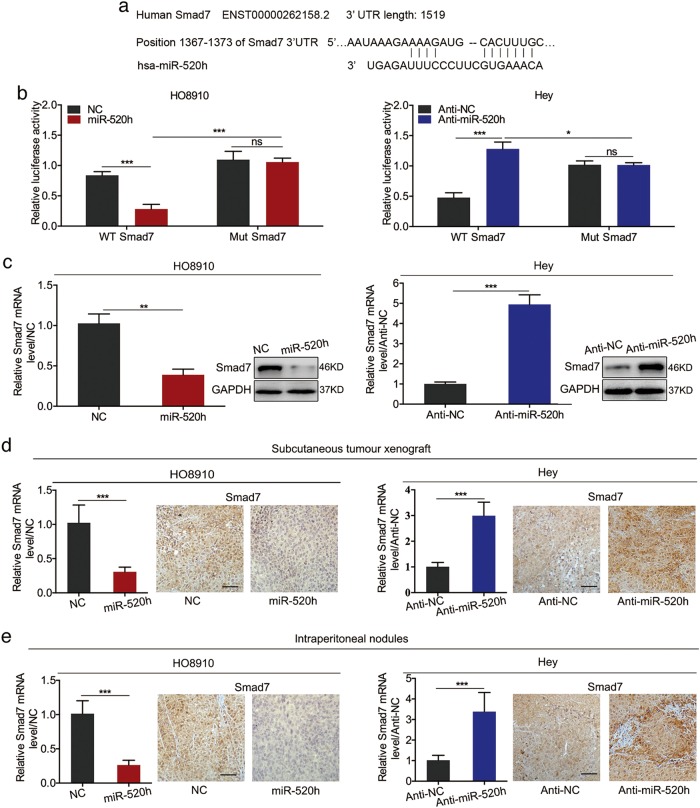
Smad7 is a miR-520h target gene. **a** The binding site of miR-520h in Smad7 3′ UTR. **b** Dual-luciferase reporter assay of HO8910 and Hey cells co-transfected with reporter plasmid containing wild-type (WT) or mutated (Mut) Smad7 3′-UTR and miR-520h mimics, anti-miR-520h, or their matched negative controls. **c** qPCR and western blotting analysis of Smad7 expression after transfection with miR-520h or anti-miR-520h. **d**, **e** Smad7 mRNA level and representative images of Smad7 IHC staining in subcutaneous tumours (**d**) and peritoneal nodes (**e**) derived from miR-520h–overexpressing HO8910 cells, miR-520h–silenced Hey cells, and their negative controls (magnification, ×200; scale bars = 50 μm). *P *<* 0.05; **P *<* 0.01; ***P *<* 0.001. NC negative control; ns not significant

To explore the mechanism(s) by which miR-520h promotes EOC metastasis, we used TargetScan (release version 7.1, http://www.targetscan.org/vert_71/) to identify metastasis-related gene targets of miR-520h. All the predicted targets are listed in Supplementary Table S7. Smad7, which could inhibit TGF-β-induced EMT^29^, was selected for further study (Fig. 3a). To determine whether Smad7 is a direct target of miR-520h, we constructed luciferase plasmids with a wild-type (WT) or mutated (Mut) Smad7 3′ UTR region and performed dual-luciferase reporter assays in cells co-transfected with WT or Mut Smad7 plasmids plus miR-520h mimics, anti–miR-520h, or their matched negative controls. Luciferase activity decreased or increased in cells co-transfected with miR-520h mimics or anti-miR-520h plus the WT Smad7 plasmid, respectively. However, no changes were observed following co-transfection with miR-520h or anti–miR-520h plus the Mut Smad7 plasmid (Fig. 3b). Notably, co-transfection with miR-520h mimics or anti-miR-520h plus the Mut plasmid reversed the changes in luciferase activity compared with co-transfection with miR-520h or anti-miR-520h plus the WT plasmid (Fig. 3b). To investigate miR-520h’s effect on endogenous Smad7, we measured Smad7 mRNA and protein levels following miR-520h overexpression or knockdown. qPCR and western blotting assays showed that, in vitro, miR-520h overexpression or knockdown suppressed or enhanced Smad7 expression, respectively (Fig. 3c). Additionally, qPCR and IHC staining in subcutaneous tumour xenografts and intraperitoneal nodules showed reduced Smad7 levels in miR-520h-overexpressing tumours, and the opposite results in miR-520-silenced tumours (Fig. 3d, e). Taken together, the in vitro and in vivo results suggested that miR-520h suppresses Smad7 in EOC cells by directly binding to its 3′ UTR region.

### miR-520h promotes EOC progression by suppressing Smad7

**Fig. 4:**
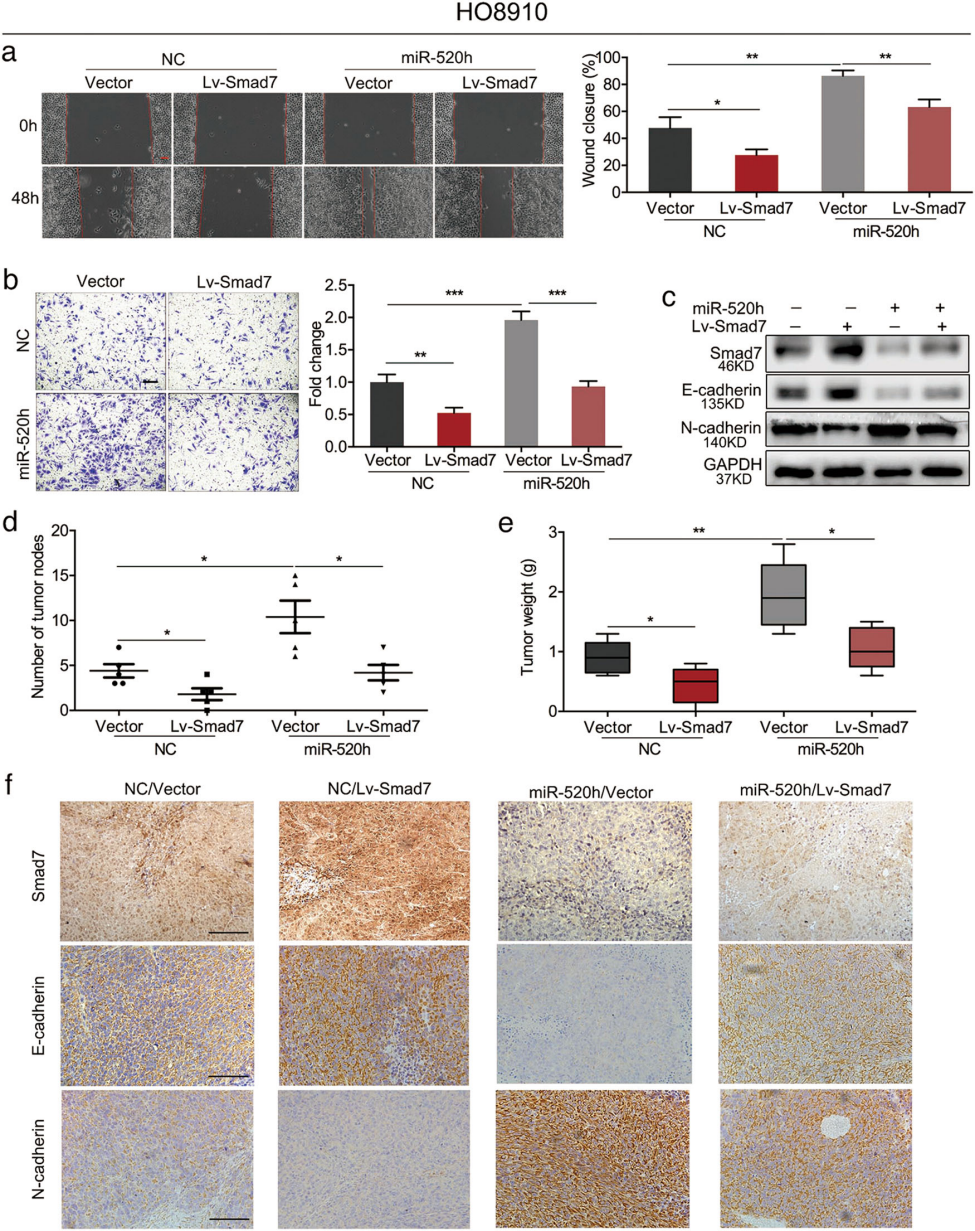
miR-520h promotes the migration and invasion of HO8910 cells in vitro, and the dissemination in vivo by inhibiting Smad7. **a**, **b** Effect of Smad7 and miR-520h overexpression on wound healing (**a**) and invasion (**b**) in HO8910 cells (magnification, ×100; scale bars = 100 μm). **c** Western blotting evaluating the effect of Smad7 and miR-520h overexpression (in HO8910 cells) on the levels of EMT markers. **d**, **e** Effect of Smad7 and miR-520h overexpression on tumour dissemination using HO8910 cells (*n* = 5). **d** The number of peritoneal tumour nodes was counted using a dissecting microscope. **e** Total tumour weights of peritoneal tumour nodes were calculated per nude mouse. **f** Representative images of Smad7, E-cadherin, and N-cadherin IHC staining in peritoneal nodes (magnification, ×200; scale bars = 100 μm). *P *<* 0.05; **P *<* 0.01; ***P *<* 0.001. NC negative control

To study the function of Smad7 in miR-520h-regulated EOC metastasis, we constructed plasmids for Smad7 overexpression or knockdown. Wound healing assays showed that Smad7 upregulation or downregulation delayed or accelerated wound closure, respectively, attenuating the effect of miR-520h deregulation (Fig. 4a; Supplementary Figure S4a). Transwell matrigel assays revealed that upregulation or downregulation of Smad7 decreased or increased the number of invading cells, respectively, and weakened the effect of miR-520h deregulation on EOC cell invasion (Fig. 4b; Supplementary Figure S4b). Moreover, Smad7 overexpression upregulated the expression of E-cadherin and downregulated that of N-cadherin, reversing the effect of miR-520h overexpression (Fig. 4c). Downregulation of Smad7 had the opposite effect (Supplementary Figure S4c). These findings showed that miR-520h promotes EOC cell migration and invasion by suppressing Smad7 in vitro.

Next, we performed in vivo experiments with cells overexpressing or silenced for miR-520h and Smad7 and their negative controls. We injected (i.p.) the cells into nude mice and measured the number and total weight of disseminated nodes per mouse. Smad7 overexpression decreased the number and total weight of disseminated nodes compared with those in the negative control, while Smad7 knockdown increased them, attenuating the miR-520h overexpression or knockdown-induced promotion or inhibition of tumour dissemination, respectively (Fig. 4d, e; Supplementary Figure S4d and S4e). IHC staining revealed that the nodules formed after injection of Smad7-overexpressing HO8910 cells had higher Smad7 and E-cadherin levels, and lower N-cadherin levels than their negative controls, partially reversing the effect of miR-520h overexpression (Fig. 4f). Conversely, the intraperitoneal nodules formed after injection of Smad7-silenced Hey cells had lower levels of Smad7 and E-cadherin and higher N-cadherin levels compared with those in their negative controls, partially attenuating the effect of miR-520h downregulation (Supplementary Figure S4f). These results suggested that miR-520h regulates EOC cell dissemination and EMT in vivo by suppressing Smad7 expression.

### miR-520h regulates EOC cell migration and invasion by activating the TGF-β signalling pathway

Smad7 is a key negative regulator of TGF-β signalling; therefore, we investigated the contribution of miR-520h to TGF-β signalling. Overexpression of miR-520h increased phosphorylated Smad2 (p-Smad2) levels and upregulated Snail expression, while miR-520h knockdown had the opposite effect (Supplementary Figure S5a). Overexpression of Smad7 partially attenuated the effect of miR-520h overexpression on p-Smad2 and Snail levels, and knockdown of Smad7 reversed the effect of miR-520h silencing (Supplementary Figure S5b). These results confirmed that miR-520h participates directly in TGF-β signalling pathway regulation.

**Fig. 5:**
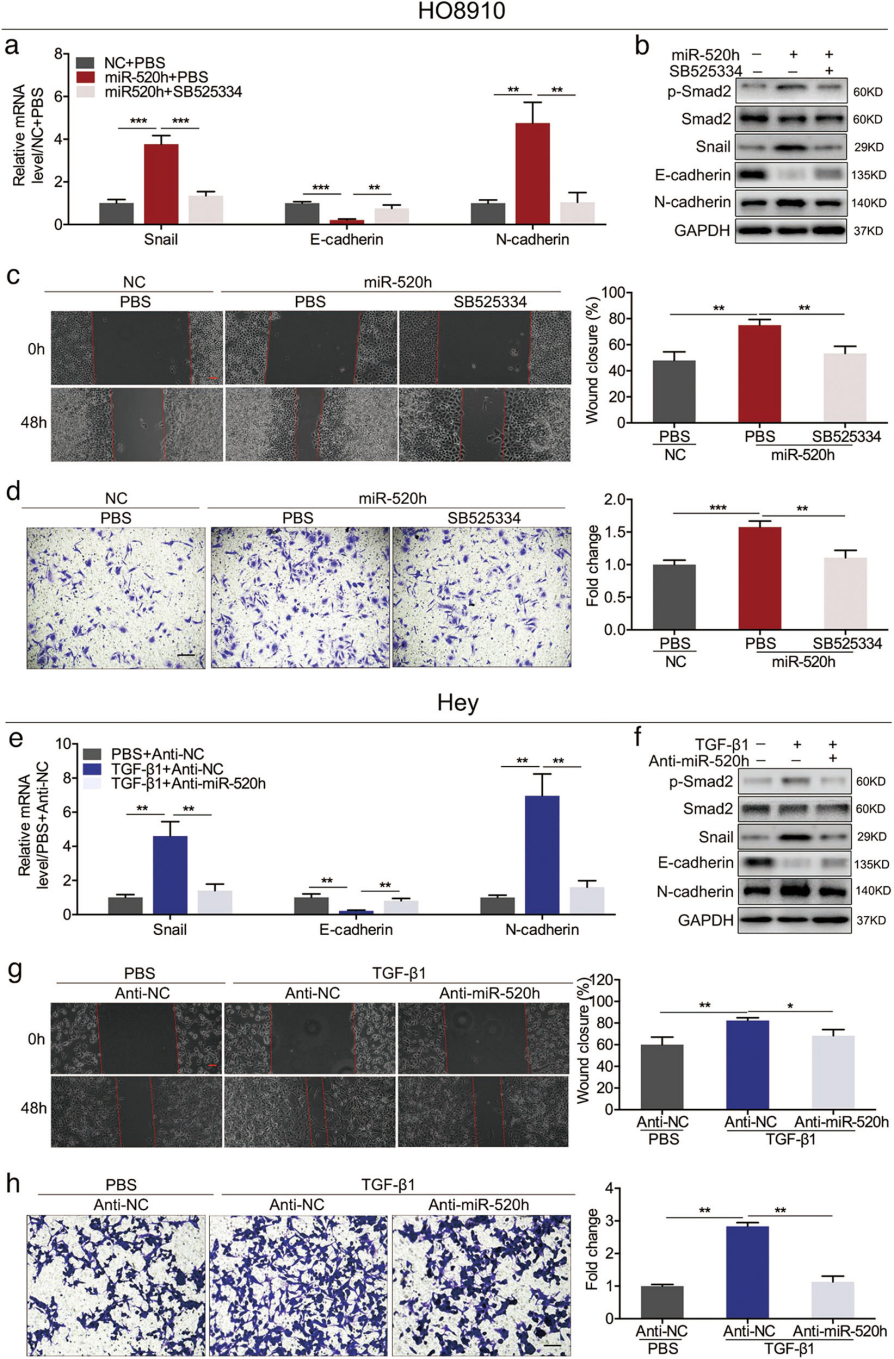
miR-520h regulates the migration and invasion of EOC cells by activating the TGF-β pathway. **a** qPCR analysis of Snail, E-cadherin, and N-cadherin mRNA levels in HO8910 cells treated with miR-520h mimics or SB525334. **b** Western blotting showing the levels of TGF-β pathway components and EMT markers in HO8910 cells treated with miR-520h mimics or SB525334. **c**, **d** Effect of SB525334 and miR-520h upregulation on cell migration (**c**) and invasion (**d**). **e** qPCR showing the levels of Snail, E-cadherin, and N-cadherin mRNAs in Hey cells treated with anti-miR-520h or TGF-β1. **f** Protein levels of components of the TGF-β pathway and EMT markers in Hey cells treated with anti-miR-520h or TGF-β1. **g**, **h** Effect of anti-miR-520h and TGF-β1 stimulation on migratory (**g**) and invasive capacities (**h**) of Hey cells. Magnification for all panels, ×100; scale bars = 100 μm. *P *<* 0.05; **P *<* 0.01; ***P *<* 0.001. NC negative control

Next, we treated miR-520h-overexpressing HO8910 cells with a specific inhibitor (SB525334) of the TGF-β/Smad7 pathway for 48 h. SB525334 partially reversed the upregulation of p-Smad2, Snail, and N-cadherin and the downregulation of E-cadherin caused by miR-520h overexpression (Fig. 5a, b). Additionally, SB525334 inhibited the migration and invasion of miR-520h-overexpressing HO8910 cells (Fig. 5c, d).

TGF-β1 promotes Smad2 phosphorylation, Snail and N-cadherin upregulation, and E-cadherin downregulation dose-dependently (Supplementary Figure S6a and S6b). We treated miR-520h-silenced Hey cells with 10 ng/ml of TGF-β1 for 48 h, and found that miR-520h knockdown significantly reversed the effect of TGF-β1 on the expression of these proteins (Fig. 5e, f). Moreover, knockdown of miR-520h greatly attenuated the effect of TGF-β1 on EOC cell migration and invasion (Fig. 5g, h). Collectively, these data indicated that miR-520h promotes EOC cell migration and invasion by activating TGF-β signalling.

### TGF-β1 upregulates miR-520h via c-Myb

**Fig. 6:**
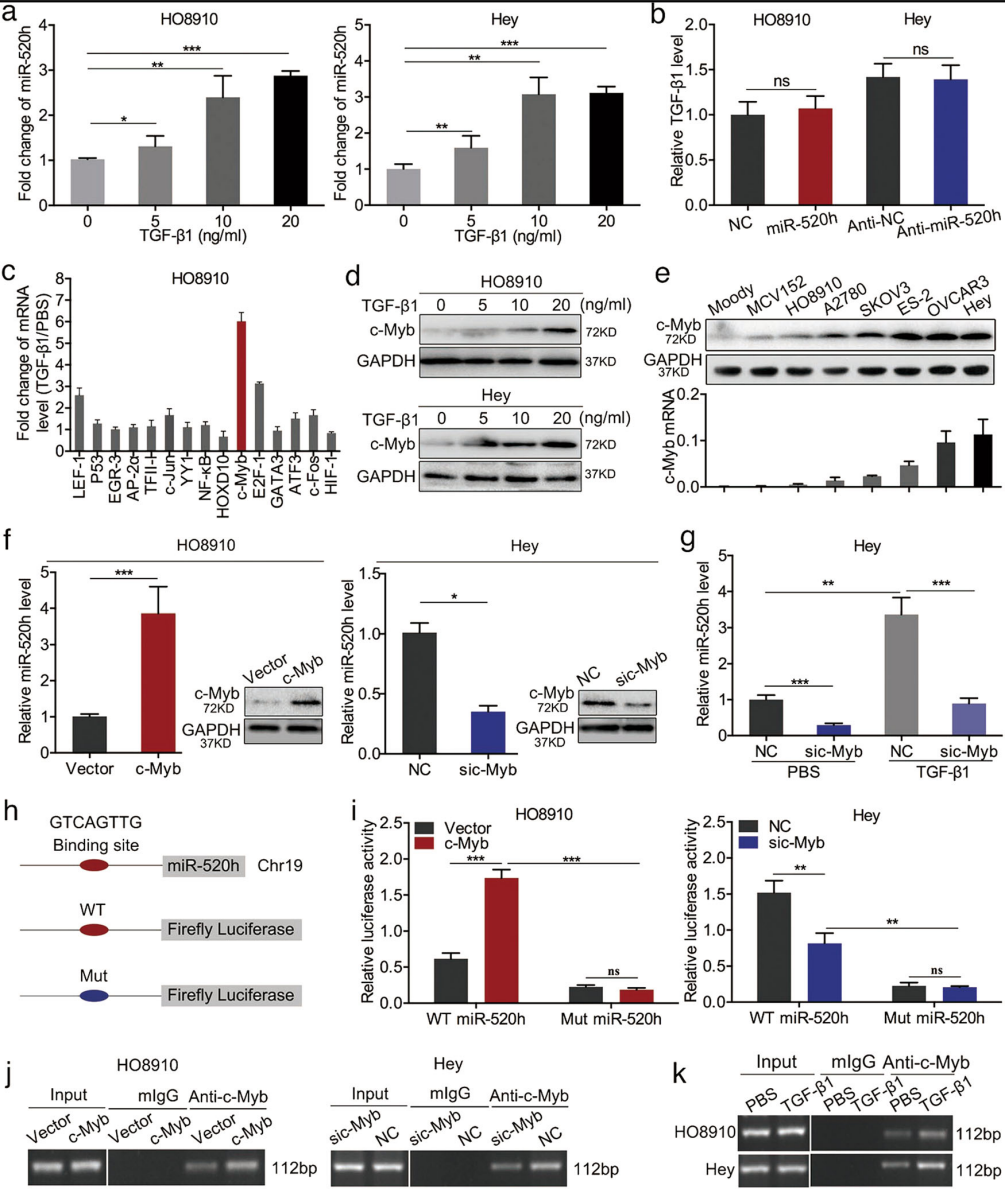
TGF-β1 activates miR-520h via c-Myb. **a** miR-520h levels in HO9010 and Hey cells treated with increasing concentrations of TGF-β1. **b** Effect of the upregulation or downregulation of miR-520h on TGF-β1 levels in the cell medium analysed using ELISA. **c** qPCR analysis showing the effect of TGF-β1 on predicted transcription factors of miR-520h. **d** c-Myb levels in cells treated with the increasing concentrations of TGF-β1. **e** Western blotting and qPCR showing the expression of c-Myb in normal (Moody), benign (MCV152), and EOC cell lines. **f** Levels of miR-520h and c-Myb after c-Myb ectopic expression or knockdown. **g** miR-520h expression levels in cells treated with TGF-β1 and c-Myb siRNA. **h** Potential c-Myb binding site in the promoter of miR-520h; a luciferase reporter plasmid was constructed containing the promoter of miR-520h with the wild-type (WT) or mutated (Mut) c-Myb binding site. **i** Dual-luciferase reporter assay showing the changes in luciferase activity in HO8910 and Hey cells co-transfected with WT or Mut luciferase reporter plasmid and c-Myb, c-Myb siRNA, or their negative controls. **j**, **k** ChIP assays in HO8910 and Hey cells treated with c-Myb overexpression plasmid, c-Myb siRNA, or their negative controls (**j**) and PBS (control) or TGF-β1 (**k**). Binding of c-Myb to the predicted site was confirmed by PCR using primers specific to the binding site. *P *<* 0.05; **P *<* 0.01; ***P *<* 0.001. ns not significant, mIgG monoclonal immunoglobulin G

To investigate the details of the crosstalk between miR-520h and the TGF-β pathway, HO8910 and Hey cells were treated with TGF-β1 for 48 h; qPCR showed that miR-520h levels were enhanced dose-dependently (Fig. 6a). The concentration of TGF-β1 in the medium of miR-520h-overexpressing HO8910 cells and miR-520h-silenced Hey cells did not fluctuate obviously compared with their negative controls (Fig. 6b), indicating that miR-520h is a downstream target of TGF-β1.

TGF-β1 could regulate the expression of its downstream genes via transcription factors (TFs)^36–38^; therefore, we searched publicly available TF datasets (PuTmiR, JASPAR, and PROMO) for potential TGF-β signalling-associated TFs that could potentially bind the miR-520h promoter, which identified 15 candidate TFs. Analysis of the mRNA expression of these TFs after stimulation of HO8910 cells with 10 ng/ml TGF-β1 for 48 h, showed that the expression of c-Myb increased the most upon treatment with TGF-β1 (by about 6-fold compared with that in untreated cells, Fig. 6c). Western blotting showed that c-Myb protein levels increased dose-dependently in both HO8910 and Hey cells after stimulation with TGF-β1 for 48 h (Fig. 6d). Meanwhile, we measured c-Myb mRNA and protein levels in a normal cell line, a benign ovarian tumour cell line, and six EOC cell lines. We found that c-Myb levels were positively correlated with those of miR-520h (Fig. 6e). Additionally, overexpression of c-Myb enhanced miR-520h expression compared with that in the negative control, while knockdown of c-Myb reduced miR-520h expression (Fig. 6f). Importantly, c-Myb knockdown significantly decreased the expression of miR-520h induced by TGF-β1 (Fig. 6g). These data indicated that c-Myb plays a significant role in TGF-β1-mediated miR-520h expression. TF datasets analysis showed that miR-520h has a putative c-Myb-binding site in its promoter (235–242, GTCAGTTG). To verify whether c-Myb upregulates miR-520h by directly binding to its promoter, we constructed a luciferase reporter vector containing a WT or Mut miR-520h promoter sequence (Fig. 6h) and performed dual-luciferase reporter assays with cells co-transfected with the reporter plasmid containing the WT or Mut miR-520h promoter vector (indicated here as WT and Mut vectors) plus a plasmid overexpressing c-Myb, a c-Myb siRNA, or their matched negative controls. Luciferase activity increased or decreased in cells co-transfected with c-Myb-overexpressing plasmid or c-Myb siRNA plus the WT vector, respectively. However, no changes were observed following co-transfection with c-Myb-overexpressing plasmid or c-Myb siRNA plus the Mut vector (Fig. 6i). Moreover, luciferase activity decreased dramatically following co-transfection with the c-Myb overexpressing plasmid plus the Mut vector compared with that in cells overexpressing c-Myb and the WT vector (Fig. 6i). Notably, chromatin immunoprecipitation (ChIP) assays revealed that c-Myb is recruited to the miR-520h promoter (Fig. 6j). Subsequently, we treated HO8910 and Hey cells with 10 ng/ml TGF-β1 for 48 h, and performed additional ChIP assays. We found that TGF-β1 enhanced the binding of c-Myb to the miR-520h promoter (Fig. 6k). We concluded that TGF-β1 upregulates miR-520h via c-Myb in EOC cells, and miR-520h plays significant roles in the development of EOC.

### Components of the TGF-β1/c-Myb/miR-520h/Smad7 axis promote EOC progress and predict poor survival

**Fig. 7:**
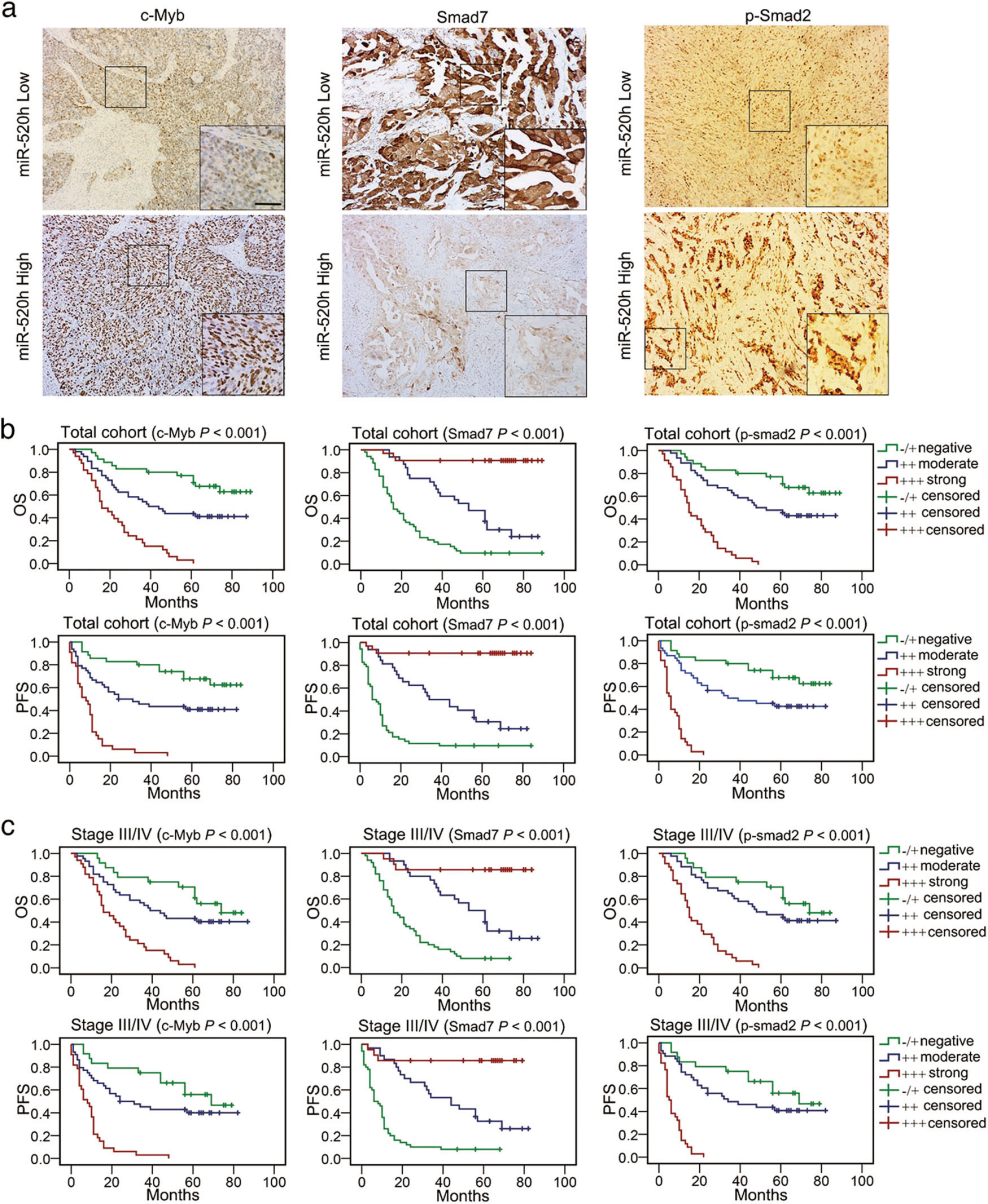
Roles of the miR-520h-associated TGF-β1/c-Myb/Smad7 axis on EOC development and prognosis. **a** Representative images of c-Myb, p-Smad2, and Smad7 IHC staining in miR-520h low or high EOC tissues (magnification, ×100; magnification inset images, ×200; scale bars = 100 μm). **b**, **c** OS and PFS analysed using the Kaplan–Meier method with log-rank testing according to c-Myb, p-Smad2, or Smad7 expression in 116 EOC cases (**b**) and 101 cases of stage III/IV EOC (**c**)

To further confirm the clinical role of miR-520h-associated activation of the TGF-β1/c-Myb/Smad7 axis in EOC, we measured the c-Myb, Smad7, p-Smad2, Snail, E-cadherin, and N-cadherin levels in 116 EOC samples and analysed the correlation between their levels and miR-520h, and their prognostic significance. miR-520h levels correlated positively with those of c-Myb, p-Smad2, Snail, and N-cadherin, and negatively with those of Smad7 and E-cadherin (*P* *<* 0.001, Supplementary Table S8; representative IHC images in Fig. 7a and Supplementary Figure S7a). Analysing the correlation between the levels of c-Myb, Smad7, p-Smad2, Snail, E-cadherin, and N-cadherin and the clinical features of the 116 patients with EOC, showed that the levels of these six proteins correlated significantly with ascites, lymph node metastasis, tumour differentiation, FIGO stage and cancer progress (Supplementary Table S9).

More importantly, high levels of c-Myb, p-Smad2, Snail, and N-cadherin indicated poorer OS and PFS in both the total cohort and stage III/IV subgroup. Conversely, high Smad7 and E-cadherin levels indicated good OS and PFS (Fig. 7b, c; Supplementary Figure S7b and S7c). These results indicated that miR-520h is a prognostic factor of EOC and that activation of the miR-520h-associated TGF-β1/c-Myb/Smad7 axis plays a key role in EOC progression and metastasis.

## Discussion

In the present study, we demonstrated that miR-520h promotes EOC progression by regulating the TGF-β1/c-Myb/Smad7 axis, activation of which predicts poor prognosis for patients with EOC. These results strongly suggested that miR-520h acts as an oncomiR in EOC^20^.

Smad7 inhibits the TGF-β/Smad signalling pathway by preventing phosphorylation of effector Smad proteins (Smad2 and Smad3) following Smad2/4 complex formation and nuclear translocation, thereby inhibiting EMT^29,39,40^. Herein, we showed that miR-520h specifically binds to the Smad7 3′ UTR and suppresses Smad7 expression, resulting in invasion and EMT of EOC cells. Moreover, Smad7 ectopic expression reversed the effects of miR-520h upregulation in vitro and in vivo, which was consistent with other reports showing that miRNAs promote cancer cells progression by downregulating Smad7^31^. Moreover, miR-520h increased the levels of p-Smad2 and Snail, subsequently stimulating TGF-β1 signalling, while miR-520h downregulation inhibited the activation of TGF-β signalling. Chemical inhibition of TGF-β signalling partially inhibited miR-520h’s effects. Similarly, miR-181a regulates TGF-β signalling by targeting Smad7 in advanced EOC^30^. Therefore, the identification and characterisation of new inhibitors of the miR-520h/Smad7/TGFβ signalling pathway might be promising to suppress EOC progression. However, more studies are needed to fully determine the effect of miR-520h deregulation in EOC.

miR-520h does not promote the progression of EOC alone; instead, it forms a positive regulatory axis with TGF-β. This is consistent with reports showing that miRNAs form regulatory networks with protein regulators to confer robustness to biological processes, and their alteration is associated with elevated risks of cell malfunction. Accordingly, clarification of the roles of miRNAs is important in the context of the regulatory networks to which they belong. In the present study, we demonstrated that, after TGF-β induction, miR-520h-mediated activation of the c-Myb/Smad7/TGF-β axis induces EOC progression. Additionally, TGF-β1 upregulates c-Myb dose-dependently, thereby upregulating miR-520h in EOC cells. This enhances the ability of EOC cells to invade and metastasize more adverse microenvironments, which explains the strong progression phenotype observed. These findings are consistent with a study in oestrogen receptor-positive breast cancer, showing that TGF-β1 induces c-Myb expression, which in turn, affects the expression of the EMT-associated genes^41^. However, the detailed mechanism of TGF-β1 upregulation of c-Myb in EOC requires further study.

Clinically, not all patients with EOC had higher miR-520h levels: miR-520h levels were higher in most (not all) of the EOC tissues (66/116); this may result from the complexity of the processes participating in the development of cancer, which involves the deregulation of many genes. Accordingly, we further explored the correlation between c-Myb, p-Smad2, Snail, and N-cadherin expression and miR-520h, and their prognostic role in patients with EOC. We found that components of the miR-520h-mediated c-Myb/TGF-β/Smad signalling axis might be suitable to predict EOC prognosis. However, additional studies exploring the potential value of miR-520h as a mechanistic prognostic biomarker, alone or in combination with other potential markers, are needed, especially for EOC cases with poor prognosis and high metastatic risk.

## Materials and methods

### Cell culture and transfection

The human EOC cell lines A2780, SKOV3, OVCAR3, ES-2, HO8910, and Hey were purchased from the Cell Bank of Chinese Academy of Sciences and cultured in RPMI 1640 (Gibco) medium with 10% fetal bovine serum (FBS, Gibco). Normal ovarian epithelial Moody cells and serous cystadenoma MCV152 cells were provided by the Shanghai General Hospital Laboratory, and cultured in minimum essential medium (MEM, Gibco) with 15% FBS. All cell lines were authenticated through short tandem repeat analysis and used within 6 months. The last authentication was on February 2017. The cell lines were maintained in an incubator at 37 °C in a humidified atmosphere with 5% CO_2_. For TGF-β1 stimulation, the cells were treated with 5, 10, or 20 ng/ml TGF-β1 (Sigma) for 48 h. To inhibit the TGF-β pathway, the cells were treated for 72 h with 10 μM SB525334 (Selleckchem).

miR-520h mimics or anti-miR-520h, lentiviral (Lv)-Smad7 or short hairpin (sh)-Smad7 plasmids, and c-Myb plasmid were constructed using the same sequences and transfected into the EOC cells as previously described^20,30,31,42^. c-Myb short interfering (si)RNA (SI00076237) and its negative control were purchased from QIAGEN and transfected using HiPerFect (QIAGEN) according to the manufacturer’s instructions.

### RNA extraction and quantitative real-time PCR (qPCR)

We have previously described the methods for total RNA extraction and amplification^23^. Supplementary Table S10 lists the primer sequences used. U6, snoRNA48, and 18S were used as the internal normalisation for miRNA quantification. mRNA data were normalised to the expression of glyceraldehyde-3-phosphate dehydrogenase (GAPDH).

### Western blotting, immunohistochemistry (IHC), and chromatin immunoprecipitation (ChIP)

The western blotting and IHC staining protocols, and the scoring method used in IHC staining, were based on our previous work^23^. Supplementary Table S11 lists the antibodies used in this study.

ChIP was performed using a ChIP assay kit (Millipore) according to the manufacturer’s instructions and with an anti-c-Myb antibody (4 μg/mg lysate). The amplified DNA was separated and semi-quantitated using agarose gel electrophoresis.

### Immunofluorescence and enzyme-linked immunosorbent assay (ELISA)

Immunofluorescence assays were performed as previously described^31^ and the images were captured with a fluorescence microscope (Leica).

ELISA was performed using a human TGF-β1 ELISA kit (R&D Systems) according to the manufacturer’s instructions.

### Cell proliferation, wound healing, and invasion assays

Cell Counting Kit-8 (CCK-8), wound healing, and Transwell assays were conducted as described previously^23^.

### Luciferase reporter assay

The dual-luciferase reporter vector containing the wild-type (WT) Smad7 3′-UTR sequence was from OriGene, and its miR-520h-binding site was mutated using a QuickChange Site-directed Mutagenesis Kit (Stratagene). The wild-type (WT) or mutated (Mut) Smad7 3′ UTR dual-luciferase reporter vectors were co-transfected together with miR-520h mimics, anti-miR-520h, or their negative controls into EOC cells using Lipofectamine 2000 (Invitrogen) according to the manufacturer’s instructions. To validate whether c-Myb binds directly to the miR-520h promoter, the miR-520h promoter sequence containing a WT c-Myb binding site was amplified and inserted into the dual-luciferase reporter vector. Alternatively, the c-Myb-binding site in the miR-520h promoter was mutated using the QuickChange Site-directed Mutagenesis Kit (Stratagene) and the generated sequence was inserted into the dual-luciferase reporter vector. Then, the WT or Mut miR-520h promoter dual-luciferase reporter vectors were co-transfected with c-Myb plasmid, c-Myb siRNA, or their negative controls into EOC cells. After 48 h, the luciferase activity was measured using a dual-luciferase reporter assay kit (Promega) and normalised to that of firefly luciferase.

### In vivo experiments

Specific pathogen-free BALB/C nude mice purchased from Shanghai Research Center for Model Organisms were used in this study. The Institutional Animal Care and Use Committee of the Shanghai General Hospital approved the in vivo assays with nude mice. The generation of subcutaneous tumours in nude mice was performed as previously reported^23^. For the dissemination experiment, 2 × 10^6^ cells were injected intraperitoneally (i.p.) into 6–8-week-old female nude mice. The mice were sacrificed after 3 weeks and the intraperitoneal nodules were counted. The nodules per mouse were harvested and weighed to calculate the total weight. Both subcutaneous tumours and intraperitoneal nodules were harvested for qPCR and IHC staining.

### Patients and tissue samples

The Institutional Research Ethics Committee of the Shanghai General Hospital approved this study. Fifteen benign ovarian tumours, seven borderline ovarian tumours, and 116 EOC human tissue samples were clinically and histopathologically diagnosed between June 2008 and June 2009 at the Department of Gynaecology and Obstetrics, Shanghai General Hospital, China. None of the 116 patients with EOC received preoperative chemotherapy. Supplementary Table S1 describes the basic clinicopathological data of the 116 patients, who were divided into two groups based on whether there was progression within 6 months after resection and adjuvant chemotherapy (the same meaning of PFS < 6 months or PFS > 6 months; clinically described as platinum resistant or platinum sensitive)^30^.

### Statistical analysis

All the data were obtained from three biological and technical repeats and reported as the mean ± SD. All statistical analyses were conducted with the SPSS 19.0 software (IBM); *P* *<* 0.05 indicated statistical significance. The difference comparisons of categorical variables were calculated using Fisher’s exact test or the chi-square test; the difference analyses of continuous variables were performed using a one-way Student’s *t-*test or analysis of variance. PFS and OS were evaluated using Kaplan–Meier analysis with log-rank testing.

## Electronic supplementary material

Supplementary Figure S1

Supplementary Figure S2-3

Supplementary Figure S4

Supplementary Figure S5-6

Supplementary Figure S7

Supplementary Table S1

Supplementary Table S2-5

Supplementary Table S6

Supplementary Table S7

Supplementary Table S8

Supplementary Table S9

Supplementary Table S10

Supplementary Table S11

supplementary figure legends
