# Supplementary figures and images for: The activation of microRNA-520h–associated TGF-β1/c-Myb/Smad7 axis promotes epithelial ovarian cancer progression
Source: Cell Death Dis. 2018 Aug 29;9(9):884. doi: 10.1038/s41419-018-0946-6 (PMC6115398; doi:10.1038/s41419-018-0946-6)

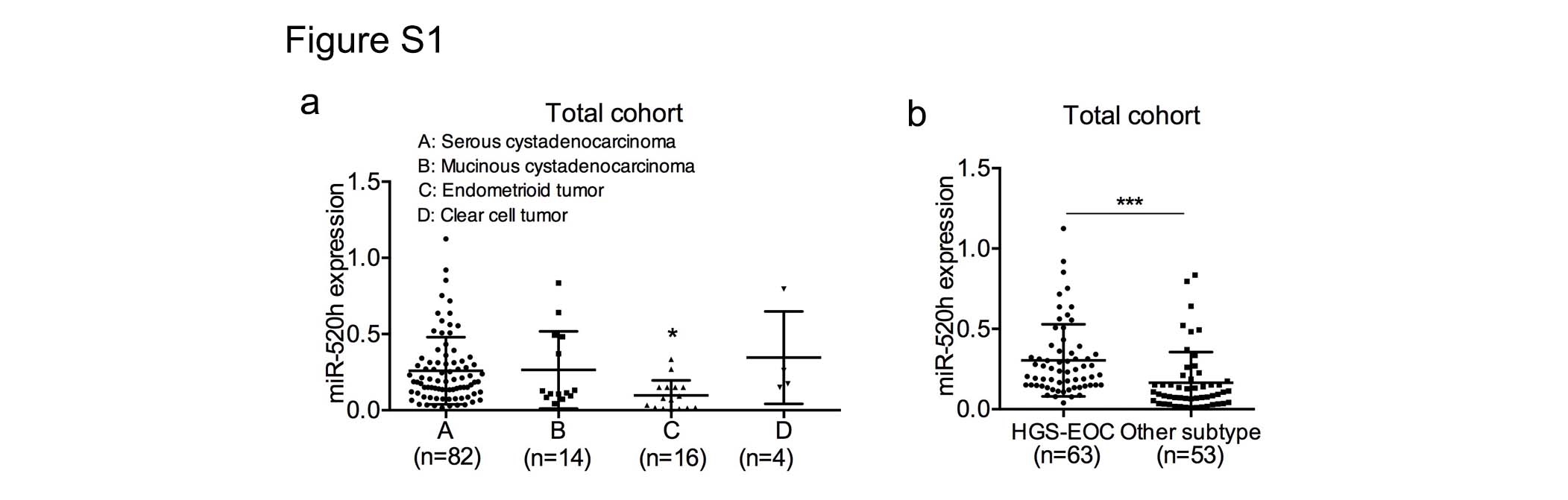

Supplement: Supplementary file 1 — Supplementary Figure S1 [file 41419_2018_946_MOESM1_ESM.jpg]

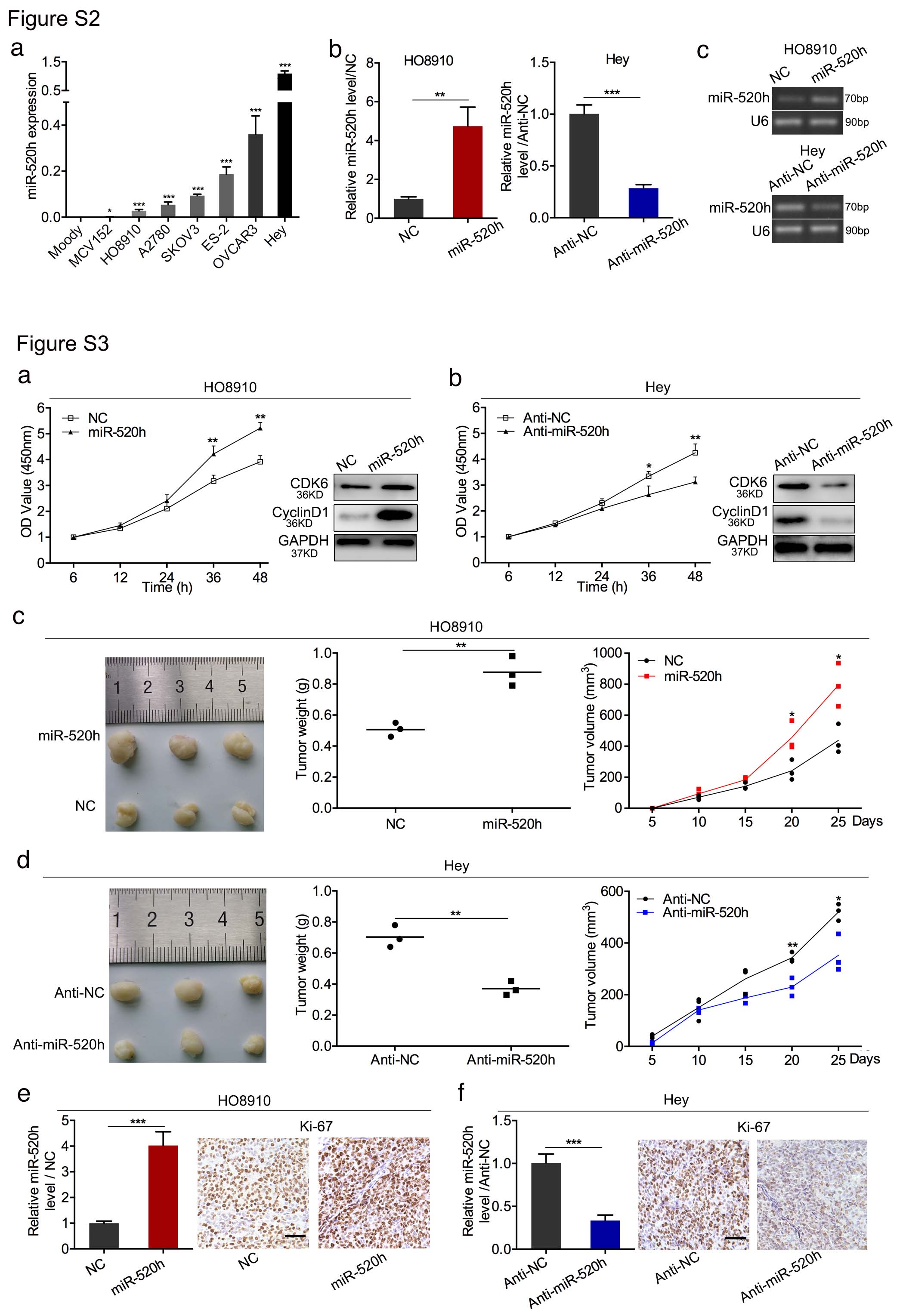

Supplement: Supplementary file 2 — Supplementary Figure S2-3 [file 41419_2018_946_MOESM2_ESM.jpg]

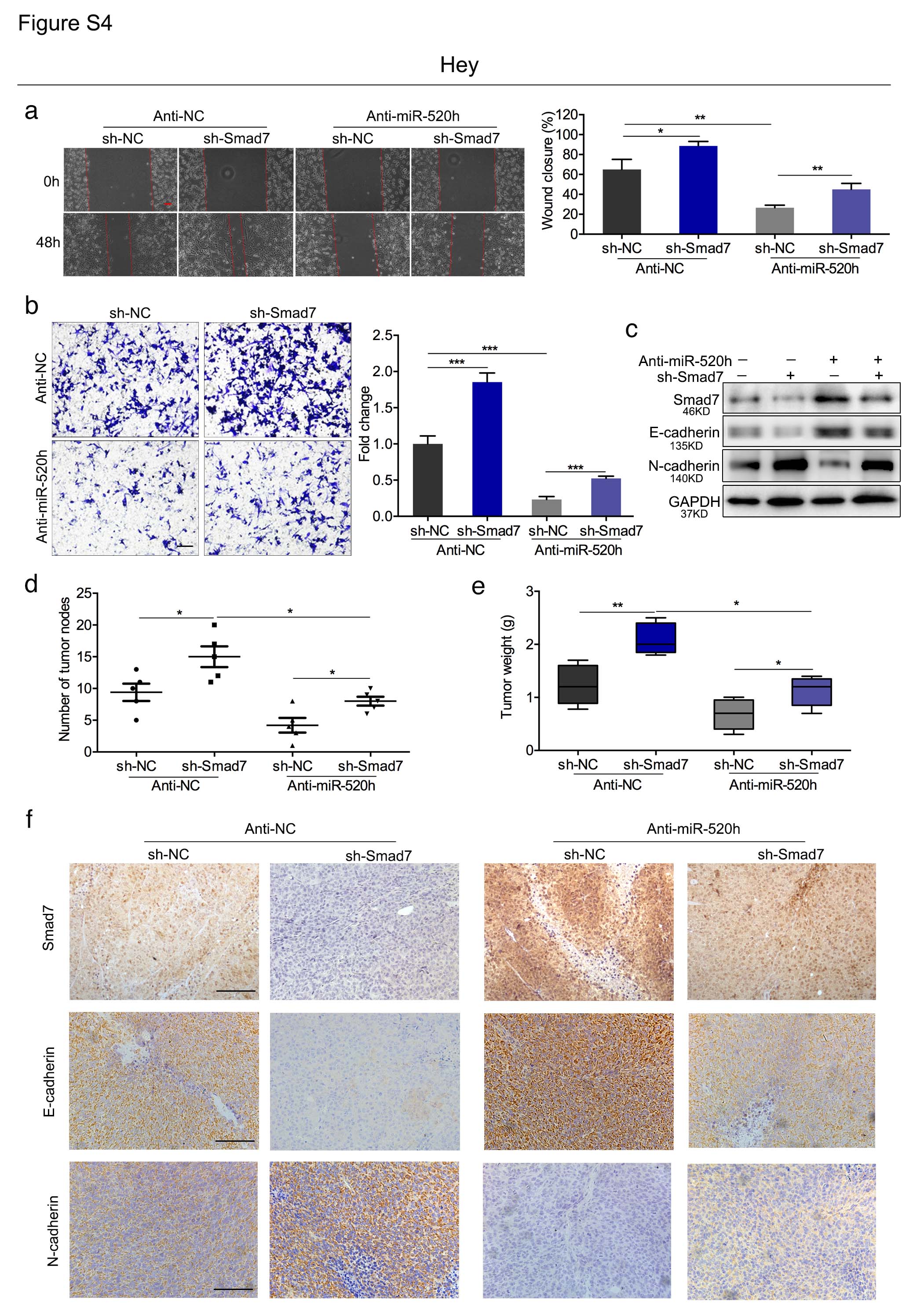

Supplement: Supplementary file 3 — Supplementary Figure S4 [file 41419_2018_946_MOESM3_ESM.jpg]

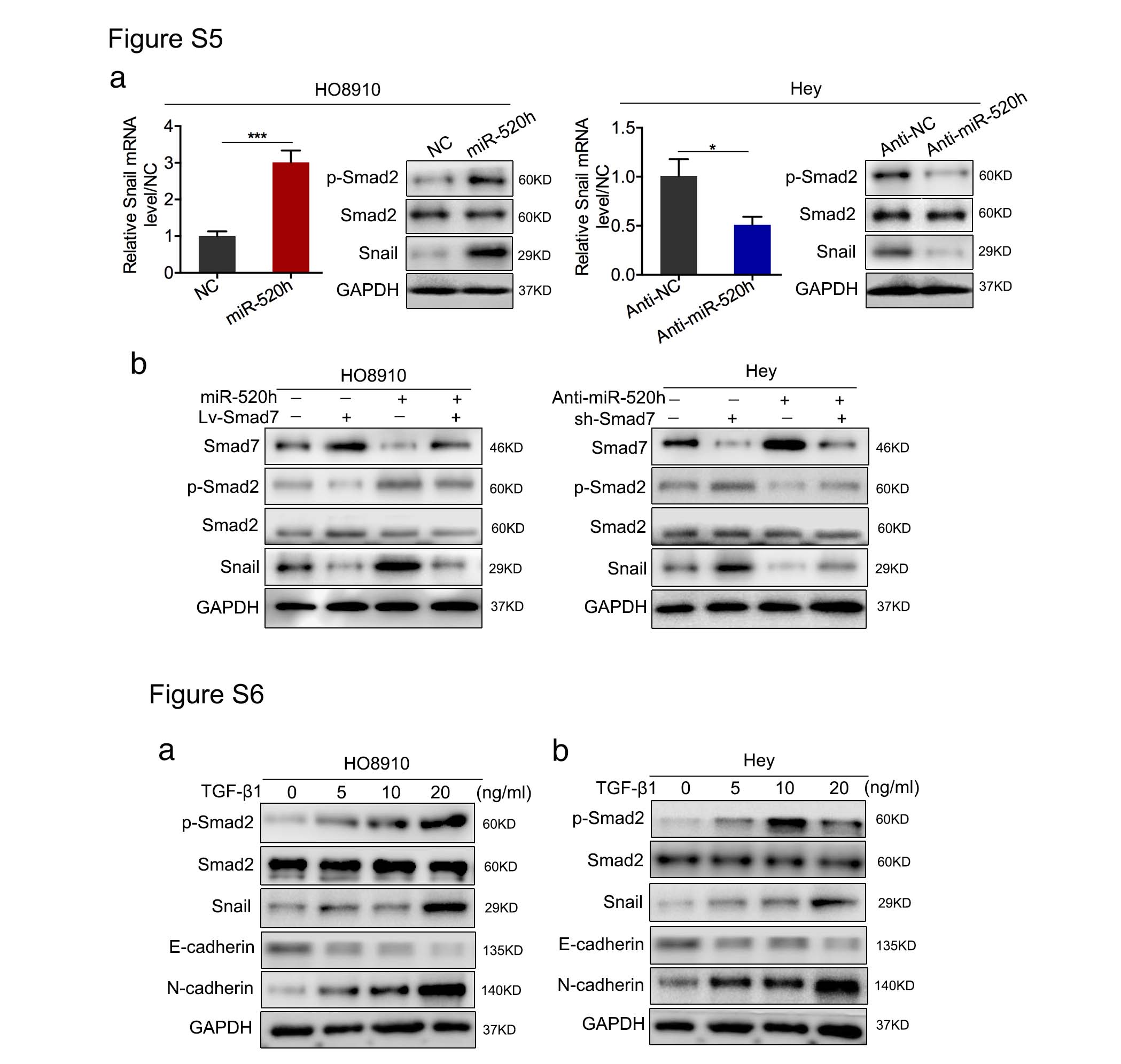

Supplement: Supplementary file 4 — Supplementary Figure S5-6 [file 41419_2018_946_MOESM4_ESM.jpg]

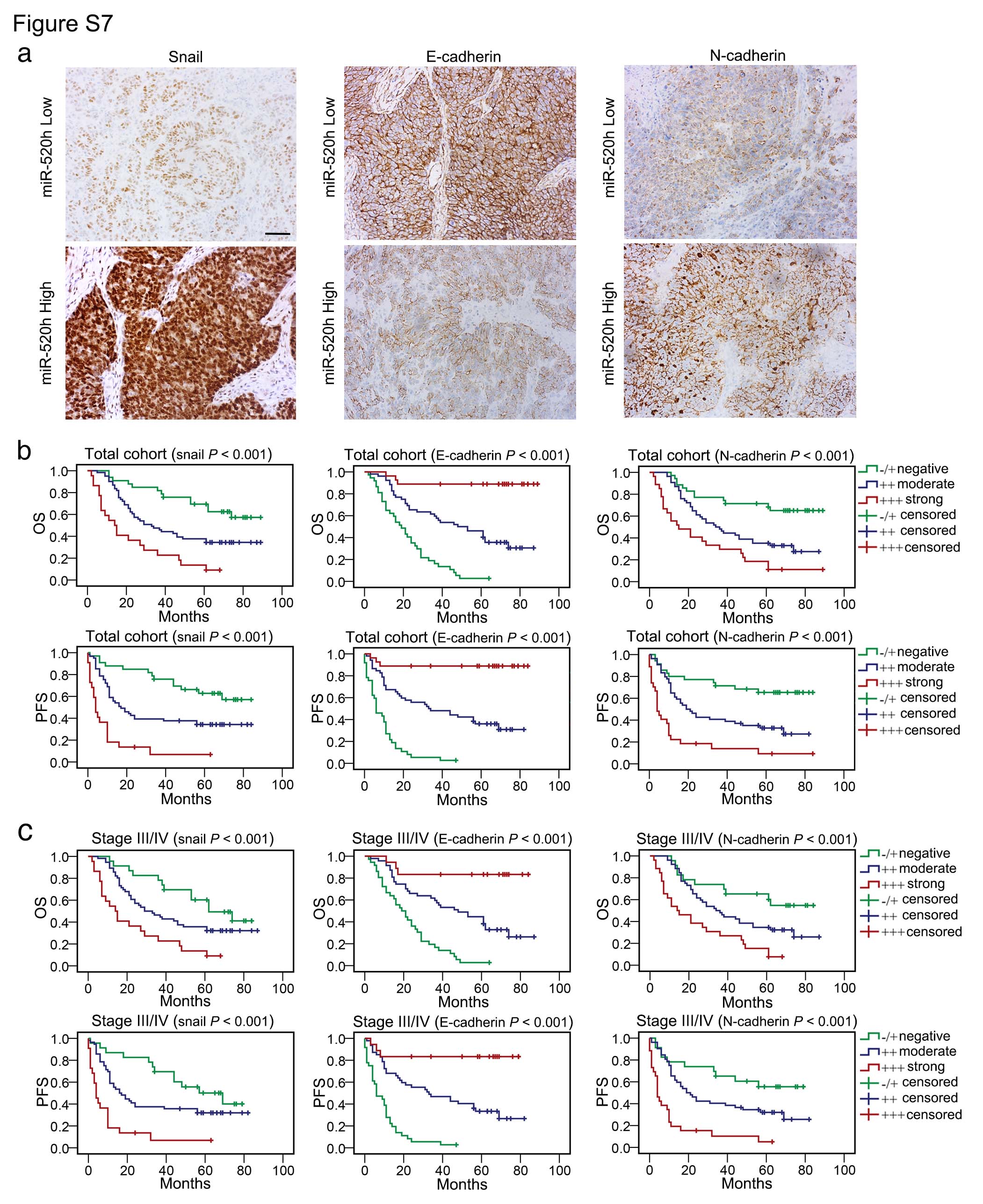

Supplement: Supplementary file 5 — Supplementary Figure S7 [file 41419_2018_946_MOESM5_ESM.jpg]
