# Supplementary material for: The activation of microRNA-520h–associated TGF-β1/c-Myb/Smad7 axis promotes epithelial ovarian cancer progression
Source: Cell Death Dis. 2018 Aug 29;9(9):884. doi: 10.1038/s41419-018-0946-6 (PMC6115398; doi:10.1038/s41419-018-0946-6)
Supplement: Supplementary file 6 — Supplementary Table S1 [file 41419_2018_946_MOESM6_ESM.docx]

**Supplementary Table S1.** Clinicopathological characteristics of the 116 patients with EOC

| **Characteristic** | **No. of patients (%)** | **Progression** | |
| --- | --- | --- | --- |
|  |  | **No, *n* = 86 (%)** | **Yes, *n* = 30 (%)** |
| Age |  |  |  |
| < 50 | 43 (37.1) | 29 (33.7) | 14 (46.7) |
| ≥ 50 | 73 (62.9) | 57 (66.3) | 16 (53.3) |
| Ascites |  |  |  |
| < 100 | 41 (35.3) | 35 (40.7) | 6 (20.0) |
| ≥ 100 | 75 (64.7) | 51 (59.3) | 24 (80.0) |
| Serum CA-125 level |  |  |  |
| < 35 | 8 (7.0) | 6 (7.0) | 2 (6.7) |
| ≥ 35 | 108 (93.0) | 80 (93.0) | 28 (93.3) |
| Lymph node metastasis |  |  |  |
| Negative | 71 (61.2) | 71 (82.6) | 0 (0.0) |
| Positive | 45 (38.8) | 15 (17.4) | 30 (100.0) |
| Tumour differentiation |  |  |  |
| G1 | 23 (19.8) | 20 (23.3) | 3 (10.0) |
| G2 | 41 (35.3) | 34 (39.5) | 7 (23.3) |
| G3 | 52 (44.8) | 32 (37.2) | 20 (66.7) |
| Histology type |  |  |  |
| Serous | 82 (70.7) | 64 (74.3) | 18 (70.7) |
| Mucinous | 14 (12.1) | 12 (14.0) | 2 (12.1) |
| Endometrioid | 16 (13.8) | 9 (10.5) | 7 (13.8) |
| Clear cell | 4 (3.4) | 1 (1.2) | 3 (3.4) |
| Residual tumour size |  |  |  |
| < 1 cm | 80 (69.0) | 67 (77.9) | 13 (43.3) |
| ≥ 1 cm | 36 (31.0) | 19 (22.1) | 17 (56.7) |
| FIGO stage |  |  |  |
| I | 4 (3.4) | 4 (4.6) | 0 (0) |
| II | 11 (9.5) | 9 (10.5) | 2 (6.7) |
| III-IV | 101 (87.1) | 73 (84.9) | 28 (93.3) |

FIGO, International Federation of Gynecology and Obstetrics.

Progression was evaluated within 6 months after surgery plus primary chemotherapy (clinically describes as platinum resistant).
