# Supplementary material for: The activation of microRNA-520h–associated TGF-β1/c-Myb/Smad7 axis promotes epithelial ovarian cancer progression
Source: Cell Death Dis. 2018 Aug 29;9(9):884. doi: 10.1038/s41419-018-0946-6 (PMC6115398; doi:10.1038/s41419-018-0946-6)
Supplement: Supplementary file 7 — Supplementary Table S2-5 [file 41419_2018_946_MOESM7_ESM.docx]

**Table S2.** The correlation between expression levels of miR-520h and PFS<6 months (platinum resistant) in 116 ovarian cancer cases (Spearman’s rank correlation)

|  | n | miR-520h | | | |
| --- | --- | --- | --- | --- | --- |
|  |  | Low (n=50) | High (n=66) | r | *P* value |
| Whole cohort |  |  |  |  |  |
| PFS < 6 | 86 | 44 | 42 | 0.276 | 0.003 |
| PFS > 6 | 30 | 6 | 24 |  |  |

**Table S3.** The correlation between expression levels of miR-520h and PFS<6 months (platinum resistant) in 15 stage I/II ovarian cancer cases (Spearman’s rank correlation)

|  | n | miR-520h | | | |
| --- | --- | --- | --- | --- | --- |
|  |  | Low (n=12) | High (n=3) | r | *P* value |
| Stage I/II |  |  |  |  |  |
| PFS < 6 | 13 | 12 | 1 | 0.784 | 0.029 |
| PFS > 6 | 2 | 0 | 2 |  |  |

**Table S4.** The correlation between expression levels of miR-520h and PFS<6 months (platinum resistant) in 106 stage III/IV ovarian cancer cases (Spearman’s rank correlation)

|  | n | miR-520h | | | |
| --- | --- | --- | --- | --- | --- |
|  |  | Low (n=38) | High (n=63) | r | *P* value |
| Stage III/IV |  |  |  |  |  |
| PFS < 6 | 73 | 32 | 41 | 0.207 | 0.042 |
| PFS > 6 | 28 | 6 | 22 |  |  |

**Table S5.** The correlation between expression levels of miR-520h and PFS<6 months (platinum resistant) in 43 high-grade serous ovarian cancer patients (Spearman’s rank correlation)

|  | n | miR-520h | | | |
| --- | --- | --- | --- | --- | --- |
|  |  | Low (n=16) | High (n=47) | r | *P* value |
| HGS-EOC |  |  |  |  |  |
| PFS < 6 | 45 | 15 | 30 | 0.288 | 0.026 |
| PFS > 6 | 18 | 1 | 17 |  |  |

PFS: progress free survival.
