# Supplementary material for: The activation of microRNA-520h–associated TGF-β1/c-Myb/Smad7 axis promotes epithelial ovarian cancer progression
Source: Cell Death Dis. 2018 Aug 29;9(9):884. doi: 10.1038/s41419-018-0946-6 (PMC6115398; doi:10.1038/s41419-018-0946-6)
Supplement: Supplementary file 8 — Supplementary Table S6 [file 41419_2018_946_MOESM8_ESM.docx]

**Supplementary** **Table S6.** Correlations between clinical features and miR-520h expression in 116 patients with EOC

| **Characteristic** | **Patients (No.)** | **miR-520h expression** | | ***P*** |  |
| --- | --- | --- | --- | --- | --- |
|  |  | **Low (*n* = 50)** | **High (*n* = 66)** |  |  |
| Age |  |  |  |  |  |
| < 50 | 43 | 18 | 25 | 0.849 |  |
| ≥ 50 | 73 | 32 | 41 |  |  |
| Ascites |  |  |  |  |  |
| < 100 | 41 | 23 | 18 | 0.037* | |
| ≥ 100 | 75 | 27 | 48 |  |  |
| Serum CA-125 level |  |  |  |  |  |
| < 35 | 8 | 4 | 4 | 0.724 |  |
| ≥ 35 | 108 | 46 | 62 |  |  |
| Lymph node metastasis |  |  |  |  |  |
| Negative | 71 | 41 | 30 | < 0.001* |  |
| Positive | 45 | 9 | 36 |  |  |
| Tumour differentiation |  |  |  |  |  |
| G1 | 23 | 17 | 6 | < 0.001* |  |
| G2 | 41 | 23 | 18 |  |  |
| G3 | 52 | 10 | 42 |  |  |
| Histology type |  |  |  |  |  |
| Serous | 82 | 31 | 51 | 0.029* |  |
| Mucinous | 14 | 9 | 5 |  |  |
| Endometrioid | 16 | 10 | 6 |  |  |
| Clear cell | 4 | 0 | 4 |  |  |
| Residual tumour size |  |  |  |  |  |
| < 1cm | 80 | 42 | 38 | 0.002* |  |
| ≥ 1cm | 36 | 8 | 28 |  |  |
| FIGO stage |  |  |  |  |  |
| I | 4 | 4 | 0 |  |  |
| II | 11 | 8 | 3 | 0.004* |  |
| III-IV | 101 | 38 | 63 |  |  |

**P* < 0.05 indicates a significant relationship among the variables.

FIGO, International Federation of Gynecology and Obstetrics.
