# Supplementary material for: The activation of microRNA-520h–associated TGF-β1/c-Myb/Smad7 axis promotes epithelial ovarian cancer progression
Source: Cell Death Dis. 2018 Aug 29;9(9):884. doi: 10.1038/s41419-018-0946-6 (PMC6115398; doi:10.1038/s41419-018-0946-6)
Supplement: Supplementary file 10 — Supplementary Table S8 [file 41419_2018_946_MOESM10_ESM.docx]

**Table S8.** The correlation between expression levels of miR-520h and c-Myb, Smad7, p-Smad2, Snail, E-cadherin, and N-cadherin in 116 ovarian cancer cases (Spearman’s rank correlation)

|  | n | miR-520h | | | |
| --- | --- | --- | --- | --- | --- |
|  |  | Low (n=50) | High (n=66) | r | *P* value |
| c-Myb |  |  |  |  |  |
| Negative | 35 | 32 | 3 | 0.594 | < 0.001 |
| Moderate | 48 | 13 | 35 |  |  |
| Strong | 33 | 5 | 28 |  |  |
| Smad7 |  |  |  |  |  |
| Negative | 52 | 9 | 43 | -0.493 | < 0.001 |
| Moderate | 32 | 17 | 15 |  |  |
| Strong | 32 | 24 | 8 |  |  |
| p-Smad2 |  |  |  |  |  |
| Negative | 35 | 32 | 3 | 0.583 | < 0.001 |
| Moderate | 46 | 12 | 34 |  |  |
| Strong | 35 | 6 | 29 |  |  |
| Snail |  |  |  |  |  |
| Negative | 33 | 28 | 5 | 0.492 | < 0.001 |
| Moderate | 61 | 18 | 43 |  |  |
| Strong | 22 | 4 | 18 |  |  |
| E-cadherin |  |  |  |  |  |
| Negative | 37 | 5 | 32 | -0.479 | < 0.001 |
| Moderate | 52 | 24 | 28 |  |  |
| Strong | 27 | 21 | 6 |  |  |
| N-cadherin |  |  |  |  |  |
| Negative | 35 | 32 | 3 | 0.492 | < 0.001 |
| Moderate | 54 | 10 | 44 |  |  |
| Strong | 27 | 8 | 19 |  |  |
