# Supplementary material for: The activation of microRNA-520h–associated TGF-β1/c-Myb/Smad7 axis promotes epithelial ovarian cancer progression
Source: Cell Death Dis. 2018 Aug 29;9(9):884. doi: 10.1038/s41419-018-0946-6 (PMC6115398; doi:10.1038/s41419-018-0946-6)
Supplement: Supplementary file 12 — Supplementary Table S10 [file 41419_2018_946_MOESM12_ESM.docx]

**Supplementary Table S10.** The primer sequences used in quantitative PCR assays

|  | Forward | Reverse |
| --- | --- | --- |
| U6 | AAAGCAAATCATCGGACGACC | GTACAACACATTGTTTCCTCGGA |
| snoRNA48 | CGGGGATAGGAAGGGAGTGA | AGCCTGAGAAATGCTGGACC |
| 18S | GGAGCCTGCGGCTTAATTTG | CCACCCACGGAATCGAGAAA |
| E-cadherin | CGAGAGCTACACGTTCACGG | GGGTGTCGAGGGAAAAATAGG |
| N-cadherin | TCAGGCGTCTGTAGAGGCTT | ATGCACATCCTTCGATAAGACTG |
| Smad7 | TTCCTCCGCTGAAACAGGG | CCTCCCAGTATGCCACCAC |
| Snail | TCGGAAGCCTAACTACAGCGA | AGATGAGCATTGGCAGCGAG |
| LEF-1 | AGAACACCCCGATGACGGA | GGCATCATTATGTACCCGGAAT |
| P53 | CAGCACATGACGGAGGTTGT | TCATCCAAATACTCCACACGC |
| EGR-3 | GACATCGGTCTGACCAACGAG | GGCGAACTTTCCCAAGTAGGT |
| AP-2α | AGGTCAATCTCCCTACACGAG | GGAGTAAGGATCTTGCGACTGG |
| TFII-I | TTGTCGTCGGAACTGAAAGAG | CGATTTGCCTGGGTTGTAGAT |
| C-Jun | TCCAAGTGCCGAAAAAGGAAG | CGAGTTCTGAGCTTTCAAGGT |
| YY1 | ACGGCTTCGAGGATCAGATTC | TGACCAGCGTTTGTTCAATGT |
| NF-κB | AACAGAGAGGATTTCGTTTCCG | TTTGACCTGAGGGTAAGACTTCT |
| HOXD10 | GACATGGGGACCTATGGAATGC | CGGATCTGTCCAACTGTCTACT |
| c-Myb | GAAAGCGTCACTTGGGGAAAA | GAAAGCGTCACTTGGGGAAAA |
| E2F-1 | CATCCCAGGAGGTCACTTCTG | GACAACAGCGGTTCTTGCTC |
| GATA3 | GCCCCTCATTAAGCCCAAG | TTGTGGTGGTCTGACAGTTCG |
| ATF3 | CCTCTGCGCTGGAATCAGTC | TTCTTTCTCGTCGCCTCTTTTT |
| c-Fos | CCGGGGATAGCCTCTCTTACT | CCAGGTCCGTGCAGAAGTC |
| HIF-1 | GAACGTCGAAAAGAAAAGTCTCG | CCTTATCAAGATGCGAACTCACA |
