# Supplementary material for: The activation of microRNA-520h–associated TGF-β1/c-Myb/Smad7 axis promotes epithelial ovarian cancer progression
Source: Cell Death Dis. 2018 Aug 29;9(9):884. doi: 10.1038/s41419-018-0946-6 (PMC6115398; doi:10.1038/s41419-018-0946-6)
Supplement: Supplementary file 13 — Supplementary Table S11 [file 41419_2018_946_MOESM13_ESM.docx]

**Supplementary Table S11.** The antibodies used in this study

| Antibody | WB | IHC | IF | Company |
| --- | --- | --- | --- | --- |
| CDK6 (#13331) | 1:1000 | - | - | Cell Signaling Technology |
| CyclinD1 (#2978) | 1:1000 | - | - | Cell Signaling Technology |
| GAPDH (ab181602) | 1:10000 | - | - | Abcam |
| E-cadherin (#3195) | 1:1000 | 1:400 | 1:200 | Cell Signaling Technology |
| N-cadherin (#13116) | 1:1000 | 1:125 | 1:200 | Cell Signaling Technology |
| Ki-67 (#9027) | - | 1:400 | - | Cell Signaling Technology |
| Smad7 (ab55493) | 1:500 | 1:100 | - | Abcam |
| Snail (sc-271977) | 1:1000 | 1:100 | - | Santa Cruz |
| Smad2 (ab40855) | 1:2000 | - | - | Abcam |
| p-Smad2 (ab188334) | 1:1000 | 1:100 | - | Abcam |
| c-Myb (ab109127) | 1:5000 | 1:50 | - | Abcam |
