# Supplementary material for: The activation of microRNA-520h–associated TGF-β1/c-Myb/Smad7 axis promotes epithelial ovarian cancer progression
Source: Cell Death Dis. 2018 Aug 29;9(9):884. doi: 10.1038/s41419-018-0946-6 (PMC6115398; doi:10.1038/s41419-018-0946-6)
Supplement: Supplementary file 14 — supplementary figure legends [file 41419_2018_946_MOESM14_ESM.docx]

**Supplementary Figure Legends**

**Fig. S1 Expression of miR-520h in EOC. a** Relative miR-520h expression in different pathological types of EOC. **b** Relative miR-520h levels in 116 EOC tissues with HGS-EOC versus the other subtypes. **P* < 0.05; ****P* < 0.001.

**Fig. S2 miR-520h levels in EOC cells. a** Relative miR-520h expression in normal (Moody), benign (MCV152), and EOC cell lines. **b** Levels of miR-520h after transfected with miR-520h mimics or anti–miR-520h. **c** Agarose gel electrophoresis of the qPCR products from (**a**). **P* < 0.05; ***P* < 0.01; ****P* < 0.001. NC, negative control.

**Fig. S3 miR-520h promotes EOC cells proliferation *in vitro* and *in vivo*. a-b** Effect of miR-520h ectopic expression (**a**) or knockdown (**b**) on cell proliferation and CDK6 and Cyclin D1 protein levels. **c-d** Effect of miR-520h overexpression (**c**) or knockdown (**d**) on the formation of subcutaneous tumour xenografts in nude mice, as compared by tumour weight and volume (*n* = 3). **e-f** miR-520h levels and representative images of Ki-67 IHC staining in subcutaneous tumours derived from miR-520h–overexpressing HO8910 cells (**e**), miR-520h-silenced Hey cells (**f**), and their negative controls (magnification, ×200; scale bars = 50 μm). **P* < 0.05; ***P* < 0.01; ****P* < 0.001. NC, negative control.

**Fig. S4 Downregulation of miR-520h inhibits the migration and invasion of Hey cells *in vitro* and EOC dissemination *in vivo* by upregulating Smad7. a-b** Effect of Smad7 and miR-520h knockdown on wound healing (**a**) and invasion (**b**) of Hey cells (magnification, ×100; scale bars = 100 μm). **c** Western blotting showing the expression of EMT markers upon downregulation of Smad7 and miR-520h. **d-e** Effect of Smad7 and miR-520h knockdown on Hey cells dissemination *in vivo*. **d** The peritoneal tumour nodes were counted using a dissecting microscope; **e** total weights of peritoneal tumour nodes were calculated per nude mouse (*n* = 5). **f** Representative images of Smad7, E-cadherin, and N-cadherin IHC staining in peritoneal nodes of nude mice (magnification, ×200; scale bars = 100 μm). **P* < 0.05; ***P* < 0.01; ****P* < 0.001. NC, negative control.

**Fig. S5 miR-520h activates the TGF-β pathway by inhibiting Smad7. a** qPCR and western blotting showing the expression of Snail and p-Smad2 and Smad2 upon miR-520h overexpression (left) or knockdown (right). **b** Effect of Smad7 and miR-520h overexpression (left) or Smad7 and miR-520h knockdown (right) on the expression of p-Smad2, Smad2, and Snail. **P* < 0.05; ***P* < 0.01; ****P* < 0.001. NC, negative control.

**Fig. S6 Role of TGF-β1 stimulation on the expression of EMT markers in EOC cells. a-b** Western blotting showing the expression levels of p-Smad2, Smad2, Snail, E-cadherin, and N-cadherin proteins upon treatment with TGF-β1 (0, 5, 10, 20 ng/ml) in HO8910 (**a**) and Hey (**b**) cells.

**Fig. S7 Expression of Snail, E-cadherin, and N-cadherin in EOC tissues and their prognostic role. a** Representative IHC staining images of Snail, E-cadherin, and N-cadherin in miR-520h low or high expression EOC tissues (magnification, ×200; scale bars = 100 μm). **b-c** Kaplan–Meier analysis of PFS and OS with log-rank testing according to Snail, E-cadherin, and N-cadherin expression in 116 cases of EOC (**b**) and 101 cases of stage III/IV EOC (**c**).
